# GHRH in diabetes and metabolism

**DOI:** 10.1007/s11154-024-09930-9

**Published:** 2024-11-19

**Authors:** Charlotte Steenblock, Stefan R. Bornstein

**Affiliations:** 1 Department of Internal Medicine III, University Hospital Carl Gustav Carus, Technische Universität Dresden, Dresden, Germany; 2 School of Cardiovascular and Metabolic Medicine and Sciences, Faculty of Life Sciences & Medicine, King’s College London, London, UK

**Keywords:** GHRH, GH, Diabetes, Obesity, Metabolism, GLP-1, IGF-1

## Abstract

Despite over a century of insulin therapy and recent advances in glucose monitoring, diabetes and its complications remain a significant burden. Current medications are not durable, with symptoms often returning after treatment ends, and responses vary between patients. Additionally, the effectiveness of many medications diminishes over time, highlighting the need for alternative approaches. Maintaining β-cell mass and promoting β-cell regeneration offer more curable treatments, while cell replacement therapies could be an option if regeneration is not feasible. For both strategies, enhancing β-cell survival is crucial. Growth hormone-releasing hormone (GHRH) was originally discovered for its ability to stimulate the production and release of growth hormone (GH) from the pituitary. Beyond the hypothalamus, GHRH is produced in peripheral tissues, with its receptor, GHRHR, expressed in tissues such as the pituitary, pancreas, adipose tissue, intestine, and liver. Several studies have shown that GHRH and its analogs enhance the survival of insulin-producing pancreatic β-cells both in vitro and in animal models. These beneficial effects strongly support the potential of GHRH agonists and antagonists for the clinical treatment of human metabolic diseases or for enhancing β-cell survival in cells used for transplantation. In the current review, we will discuss the roles of hypothalamic and extrahypothalamic GHRH in metabolism in physiological and pathological contexts, along with the underlying mechanisms. Furthermore, we will discuss the potential beneficial effects of GHRH analogs for the treatment of metabolic diseases.

## Introduction

The number of individuals worldwide with metabolic diseases like diabetes and obesity has been steadily rising over the past few decades and continues to grow [1]. A number of pharmaceutical treatments are available for these patients and at the same time, major advances in e.g., glucose monitoring and other artificial intelligence-based technologies have occurred [2–5]. Despite this, mimicking islet function and complex physiological feedback remains challenging. Current treatments show variable effects, are not durable and their effectiveness might diminish over time [6, 7]. Therefore, there is an unmet medical need and alternative strategies and/or development of new treatments are still required. Recent research in cell replacement strategies for treating type 1 diabetes has expanded beyond auto-, allo-, and xenotransplantation to include the differentiation of induced pluripotent stem cells into insulin-producing β-cells for transplantation [6, 8, 9]. Furthermore, gene therapy for e.g. restoration of insulin-production, is being investigated [10, 11].

Another approach is to develop new pharmaceutical strategies, such as growth hormone releasing hormone (GHRH) analogs, which have shown very promising results in animal models and in vitro cell cultures in different endocrine and metabolic research studies. However, to date, only one GHRH analog, tesamorelin, has been approved for clinical use.

Around 60 years ago, it was suggested that a factor in hypothalamic extracts could stimulate the production and release of growth hormone (GH) from the pituitary gland [12–14]. However, it was not until the 1980s that GHRH was first isolated from human pancreatic tumors causing acromegaly and subsequently characterized [15, 16]. In addition to the hypothalamus, GHRH is expressed in various other tissues, suggesting that it functions not only within the neuroendocrine axis but also in the autocrine and paracrine systems of extrahypothalamic tissues [17]. This is supported by the fact that the receptor for GHRH, GHRHR, which is a class B G protein-coupled receptor [18], is expressed mainly in the pituitary somatotroph cells, but also in extrapituitary tissues [17].

In this Review, we first outline the role of GHRH in the GHRH/GH/insulin-like growth factor-1 (IGF-1) axis and its extrapituitary functions. We then focus on the role of GHRH in type 1 and type 2 diabetes, as well as metabolic diseases. Additionally, we explore the diverse functions of synthetic GHRH agonists and antagonists in both physiology and pathology, with a particular emphasis on their therapeutic potential in metabolic disorders.

## The GHRH/GH/IGF-1 axis

GHRH is part of the neuroendocrine system, which is a complex regulatory network that acts as an interface between the nervous system (both central and peripheral) and the endocrine system. The neuroendocrine system regulates various functions, including metabolism, reproduction, blood pressure, mood, stress, thermoregulation, sleep, and the balance of body fluids and electrolytes [19]. The central nervous system (CNS) either directly engages in endocrine activities or manages endocrine cells by releasing neurotransmitters from the hypothalamus into the hypothalamic-pituitary portal circulation [20]. These neurotransmitters influence the secretory actions of the anterior pituitary gland, which in turn affects endocrine glands throughout the body.

**Fig. 1:**
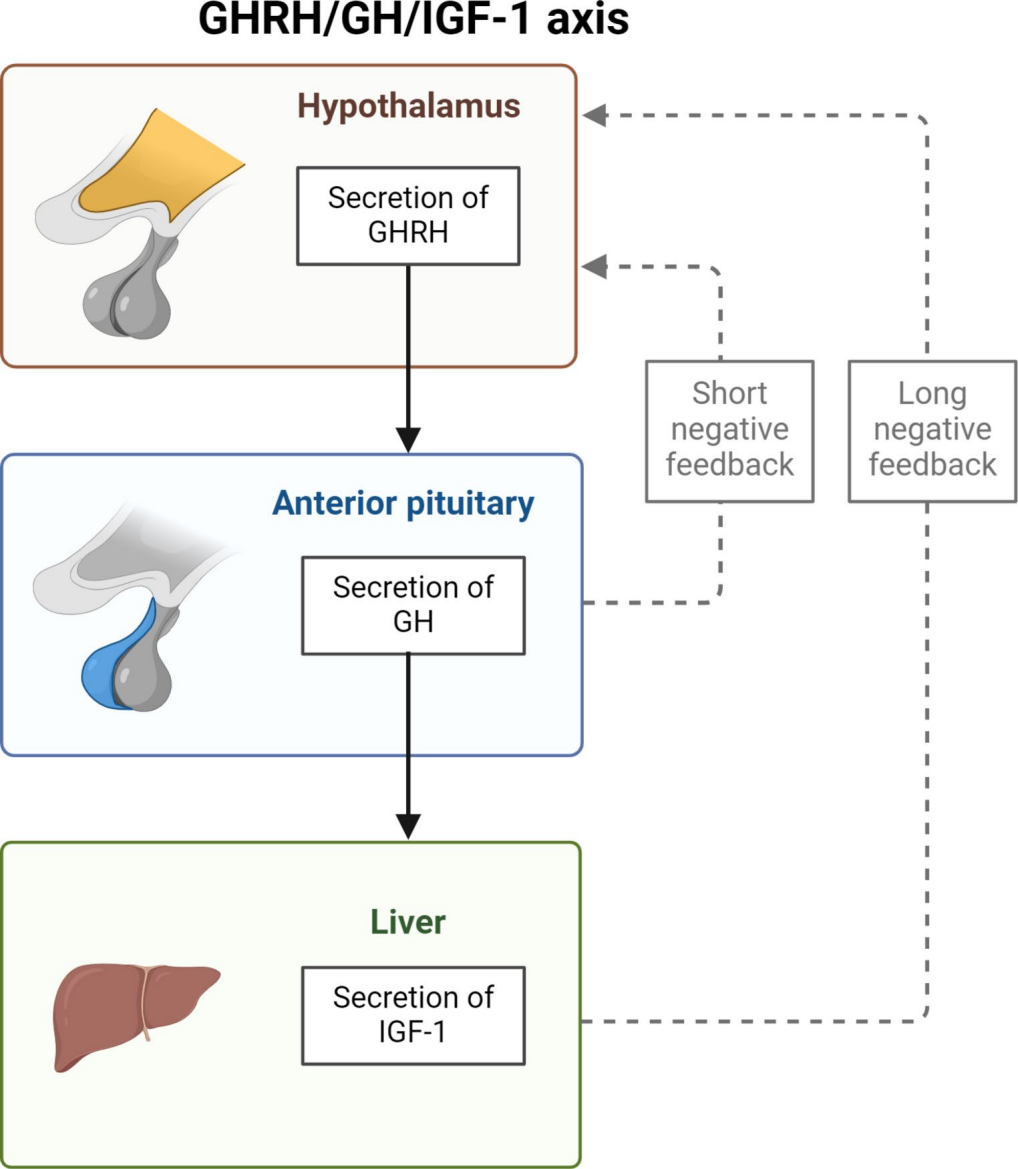
The GHRH/GH/IGF-1 axis. Simplified schematic summary of the major molecules composing the GHRH/GH/IGF-1 axis. GHRH released from the hypothalamus stimulates the release of GH from the anterior pituitary which in turn stimulates the release of IGF-1 from the liver. Feedback mechanisms of GH and IGF-1 regulate the secretion of GHRH. Created with BioRender.com

GHRH belongs to a peptide family of neurotransmitters including secretin, glucagon, glucagon-like peptides (GLPs), pituitary adenylate-cyclase-activating polypeptide (PACAP), and vasoactive intestinal polypeptide (VIP) [21–23]. GHRH is secreted by the hypothalamus, initiating a cascade of sequential events (see simplified overview in Fig. 1). GHRH reaches the pituitary gland through the hypothalamic-hypophyseal portal system of the pituitary stalk, where it binds to its receptor, GHRHR. This binding stimulates the release of GH from somatotrophs in the anterior pituitary via the Gαs subunit and cyclic adenosine monophosphate (cAMP)-dependent pathways [18, 21, 24, 25]. In addition to regulating GH, GHRH promotes somatotroph proliferation and is crucial for their postnatal expansion [24].

GH is a key regulator of somatic growth and, as a pleiotropic hormone, it also plays a role in regulating several vital processes, including nutrition, reproduction, physical activity, neuroprotection, immunity, and osmotic pressure [25]. In addition to GHRH, the secretion of GH is positively regulated by several endogenous factors, such as ghrelin, primarily produced by endocrine cells of the stomach, PACAP, and serotonin, and is inhibited by factors like the hypothalamic peptide somatostatin and catecholamines [25]. Additionally, GH secretion is regulated by thyroid hormones, leptin, and sex hormones such as androgens and estrogen, contributing to distinct sex-specific secretion patterns [26]. Other key factors influencing GH secretion include nutrition, exercise, body composition, and the onset of deep sleep [27–29]. Furthermore, a number of synthetic GH secretagogues have been shown to influence the synthesis and/or release of GH by somatotrophs in vertebrates [30].

Among its various functions, the primary action of pituitary-derived GH is to circulate to the liver, where it promotes the synthesis of insulin-like growth factor 1 (IGF-1) [31, 32]. This occurs when GH binds to its receptor, GHR, which then recruits the non-receptor protein tyrosine Janus kinase 2 (JAK2) [33]. JAK2 phosphorylates several tyrosine residues on the intracellular domain of GHR, facilitating the recruitment of other signaling molecules, including JAKs, signal transducers and activators of transcription (Stats), the mitogen activated protein kinase (MAPK) pathway, and the phosphatidylinositol 3’-kinase (PI3K) pathway [34].

IGF-1 exerts its effects by binding to and activating the IGF-1 receptor (IGF-1R), a widely expressed cell-surface tyrosine kinase receptor [35]. This results in the autophosphorylation of IGF-1R, which activates several signaling pathways, including PI3K and MAPK [36]. The ability of IGF-1 to bind to and activate IGF-1R is regulated by a family of six IGF-binding proteins (IGFBPs), which serve as transporters for IGF-1 in the bloodstream, decreasing the amount of free IGF-1 and extending its half-life [37]. IGF-1 plays a crucial role in growth and development, as well as in regulating various physiological processes. Both IGF-1 and GH exert negative feedback on GH secretion from the pituitary [38].

In addition to its production in the hypothalamus and its systemic role in the GHRH/GH/IGF-1 axis, GHRH is an important local autocrine-paracrine regulator of cellular functions in various cells and organs. GHRHR is expressed in several organs outside the pituitary, e.g. adrenal cells [39], pancreatic β-cells [40, 41], and adipocytes [42]. Additionally, different splice variants (SVs) of GHRHR have been identified in human extrapituitary cells, with SV1 being the major form [43, 44].

### Type 1 diabetes and GHRH

Type 1 diabetes is caused by the autoimmune destruction of pancreatic insulin-producing β-cells. The disease seems to result from a complex interaction between genetic predisposition, the immune system, and environmental factors [45], such as viral infections, which have been linked to the development of disease [46, 47]. Specifically, enteroviruses and coronaviruses have been shown to damage β-cells [48–50]. Although the exact mechanism by which viruses contribute to type 1 diabetes is not fully understood, it is proposed that they trigger and sustain autoimmunity, induce inflammation, and exacerbate β-cell dysfunction and stress [51].

The preservation of β-cells is crucial in both type 1 and type 2 diabetes as β-cell dysfunction, reduced mass, and apoptosis are central to insufficient insulin secretion in both types [52]. In newly diagnosed type 1 diabetes, there is a high degree of heterogeneity in the rate of β-cell loss. A recent multi-omics analysis has identified features that differ between individuals with either slow or rapid β-cell loss [53]. In the future, these differing features could potentially be used to tailor treatments for patients.

The autoimmune inflammatory process responsible for early-life islet function destruction also predisposes these patients to various other complications including hypoglycemia unawareness, peripheral neuropathy, and conditions affecting the heart, kidneys, and retinas [7]. Therefore, managing and treating type 1 diabetes is crucial.

The GHRH/GH/IGF-1 axis influences metabolic processes, including glucose and lipid metabolism. On one hand, GH promotes hepatic gluconeogenesis and lipolysis, temporarily increasing glucose and free fatty acid levels, thereby reducing insulin sensitivity [54–56]. This effect is particularly noticeable during periods of increased GH secretion, such as fasting or exercise where hypoglycemia stimulates GH release [29, 57, 58]. Under normal circumstances, plasma glucose levels are maintained within a narrow range. In healthy individuals, hypoglycemia rapidly triggers the counterregulatory response to restore blood glucose [59]. This response occurs through direct actions of low glucose on peripheral organs, such as the pancreas, and indirectly via the CNS. On the other hand, IGF-1, produced in response to GH, counteracts this effect by enhancing insulin sensitivity, contributing to glucose uptake and utilization by muscle cells and adipocytes, thus playing a role in maintaining blood sugar levels [36, 60].

**Fig. 2:**
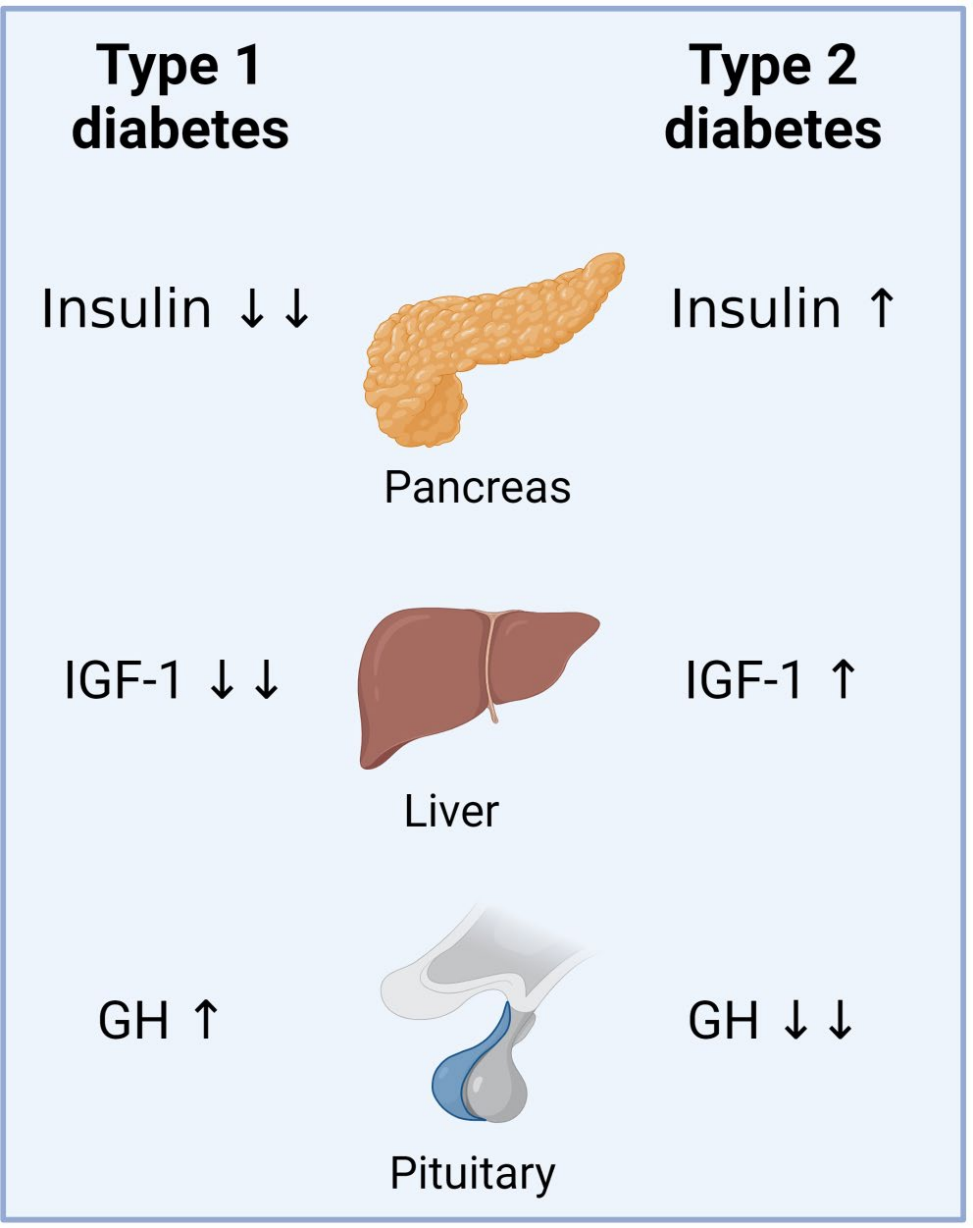
Dysregulation of GHRH in type 1 and type 2 diabetes. In type 1 diabetes, very low insulin levels lead to decreased liver sensitivity to GH. As a result, IGF-1 levels drop, and the lack of negative feedback from IGF-1 on the hypothalamus causes increases in both GHRH and GH levels. In type 2 diabetes, insulin levels are elevated to compensate for insulin resistance, which increases liver sensitivity to GH and leads to increased IGF-1 levels. Due to the negative feedback from IGF-1 on the hypothalamus, GHRH and GH levels may decrease. Created with BioRender.com

In contrast, elevated blood glucose suppresses GH secretion by promoting the release of somatostatin from the hypothalamus, which in turn inhibits GH secretion from the anterior pituitary gland [61]. This is part of a feedback loop where high glucose reduces the body’s need for additional glucose-raising hormones like GH. This tight regulation results in an overall balanced effect on glucose metabolism.

In patients with type 1 diabetes, GH secretion can become dysregulated as elevated glucose levels and reduced insulin impair normal feedback mechanisms (Fig. 2). Studies have shown that the GHRH response to a mixed meal is blunted in these patients compared to healthy controls [62]. Conversely, hypersecretion of GH in type 1 diabetes has been associated with increased circulating levels of glucose and lipids [63–66].

Previous studies have shown that GHRH neurons form a distinct subpopulation of GABAergic neurons in the hypothalamic arcuate nucleus that express glucokinase and are relatively homogeneous, with over 80% being inhibited by glucose in ex vivo electrophysiological studies [67]. GHRH neurons are activated by glucose deprivation in vivo and provide polysynaptic inputs directly to the pancreas [68].

In summary, both glucose and insulin are key regulators in the balance of GHRH and GH release, acting through complex feedback loops to modulate energy metabolism, inflammation and growth. IGF-1 and insulin can bind to and activate each other’s receptors, though with lower affinity, highlighting the close interaction between GH, IGF-1, and insulin [69]. This underscores the significance of both hypothalamic and extrahypothalamic GHRH in type 1 diabetes [56].

### Type 2 diabetes and GHRH

Type 2 diabetes accounts for 90% of all diabetes cases and is a polygenic metabolic disorder influenced by environmental factors. It is characterized by insulin resistance in peripheral tissues and impaired insulin secretion due to progressive pancreatic β-cell dysfunction and loss of β-cell mass [70, 71]. Recent research indicates that insulin-producing pancreatic β-cells do not just undergo apoptosis as previously believed, but that they also degranulate, transdifferentiate or dedifferentiate into non-functional endocrine progenitor-like cells, ultimately leading to β-cell dysfunction [72–74]. A number of studies indicate that the loss of β-cells is not an irreversible process [75]. For example, intensive insulin therapy in patients with newly diagnosed type 2 diabetes has been shown to achieve long-term remission in approximately 50% of cases [76]. Furthermore, early insulin intervention coupled with metformin has been shown to improve β-cell function resulting in a superior and longer lasting glycemic and lipid control [77].

For type 2 diabetes, lifestyle modifications such as weight loss and exercise are key to enhancing insulin sensitivity and preserving β-cell function. Additionally, various pharmacological approaches, including cytokine inhibitors and protein kinase inhibitors, are being studied to protect β-cells from inflammation and glucotoxicity [52].

Bariatric surgery has been shown to be more effective than conventional medical therapy for long-term control of type 2 diabetes in patients with severe obesity. It promotes β-cell survival and function, improves insulin sensitivity, modulates gut hormones, and increases β-cell mass, resulting in diabetes remission and better glycemic control in this subgroup of patients with type 2 diabetes [78–80]. Bariatric surgery alters the gut hormone profile, including increased glucagon-like peptide 1 (GLP-1) and peptide YY (PYY) secretion. GLP-1 is known to enhance β-cell survival and function while promoting β-cell proliferation [81]. The upregulation of GLP-1 and PYY levels after bariatric surgery contributes to improved glycemic control and β-cell preservation.

**Fig. 3:**
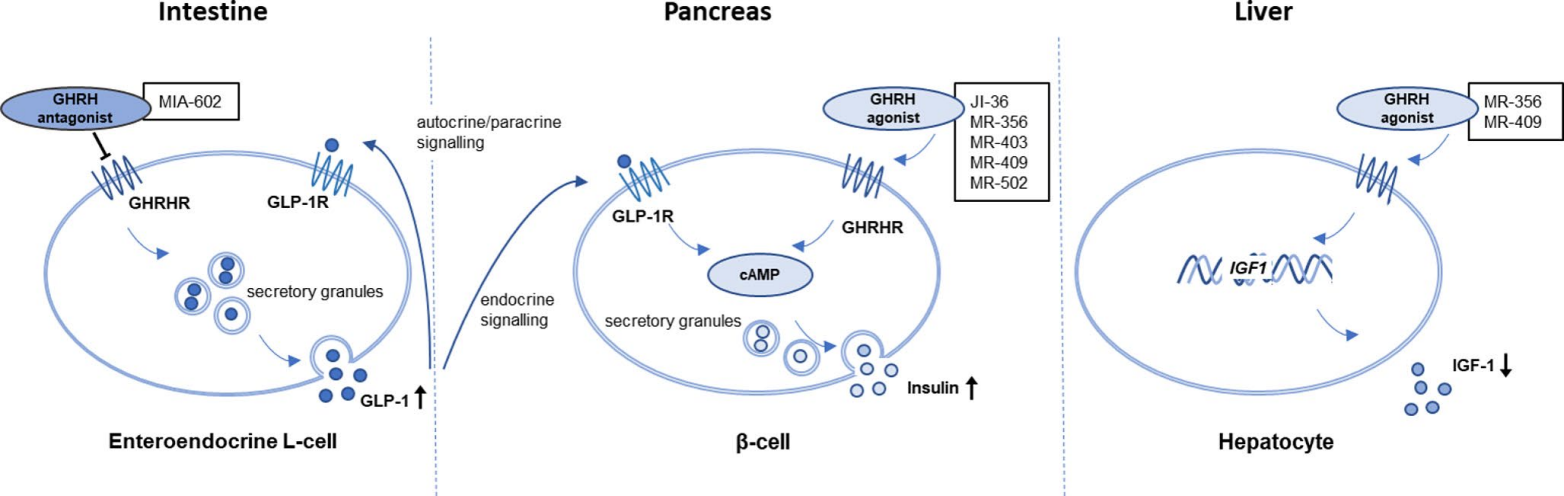
Extrapituitary metabolic effects of GHRH and its analogs. Simplified schematic summary of the extrapituitary effects of GHRH. GLP-1 is released from enteroendocrine L-cells in the intestine, stimulated by the GHRH antagonist MIA-602. In β-cells, GHRH and GHRH agonists increase insulin secretion through cAMP-mediated mechanisms. The GLP-1R-mediated survival and insulinotropic functions of GLP-1 and GLP-1 agonists involve the same signaling pathways as GHRH agonists. In the liver, IGF-1 production decreases with the addition of GHRH agonists. These are all effects that might enhance β-cell survival and insulin secretion. GHRH-R, GHRH-receptor; GLP-1, glucagon-like peptide 1; GLP-1R, glucagon-like peptide 1 receptor; IGF-1, insulin-like growth factor-1

In recent years, GLP-1 receptor (GLP-1R) agonists have proven highly effective in the treatment of obesity and type 2 diabetes [82]. These agonists contribute to increased insulin secretion in response to hyperglycemia, suppression of glucagon secretion under hyper- or euglycemic conditions, delayed gastric emptying to prevent substantial post-meal glycemic spikes, and a decrease in calorie intake and body weight [83]. GHRH and the incretin GLP-1 belong to the same class of structurally related hormones activating class B G-protein coupled receptors and operating through cAMP signaling (Fig. 3). Therefore, if GLP-1 signaling is impaired, which can occur in both type 1 and type 2 diabetes [84–89], GHRH could serve as a potential alternative treatment option.

GHRH is intricately involved in metabolic processes, and its dysregulation can lead to or exacerbate various metabolic disorders, including obesity, type 2 diabetes, cardiovascular diseases, metabolic syndrome, and growth hormone deficiency (GHD). GHRH is involved in the regulation of insulin secretion from the pancreas and abnormal GHRH signaling can impair insulin secretion, leading to hyperglycemia and contributing to the development or worsening of diabetes (Fig. 2) [90].

As mentioned above, GHRH, through its effect on GH, influences glucose metabolism. GH can increase glucose production in the liver and reduce glucose uptake in peripheral tissues, which can worsen hyperglycemia in the context of type 2 diabetes [90, 91]. In the pancreas, GHRH can enhance insulin secretion from β-cells, which is crucial for glucose uptake and regulation [22]. In the heart, extrahypothalamic GHRH has been shown to play a role in cardioprotection and energy metabolism [92, 93]. Thus, understanding and targeting GHRH signaling could provide therapeutic avenues for managing type 2 diabetes and associated comorbidities.

## Obesity, metabolic disorders and GHRH

Metabolic syndrome is a cluster of conditions, including obesity, insulin resistance, dyslipidemia, and hypertension. GHRH/GH dysregulation can contribute to each of these conditions. For example, low GH levels can lead to increased fat mass, insulin resistance, and an unfavorable lipid profile, all of which are components of metabolic syndrome [94]. Metabolic syndrome is often accompanied by chronic low-grade inflammation. GHRH has been shown to have immunomodulatory effects, which can indirectly influence metabolism. The immune system and metabolism are closely linked, and GHRH’s role in immune cells might impact metabolic responses, especially during inflammation or infection [95, 96]. Dysregulation in GHRH signaling can exacerbate inflammation, further driving metabolic dysfunction [21, 97]. For example, GHRH has been demonstrated to play a role in multiple sclerosis and its representative animal model, experimental autoimmune encephalomyelitis [98, 99]. Furthermore, GHRH antagonists have been shown to inhibit SARS-CoV-2-induced inflammation of human macrophages and peripheral blood mononuclear cells in vitro [100].

WHO reports indicate that in 2022, 43% of adults aged 18 and older were overweight, while 16% were living with obesity. These figures continue to rise, highlighting that obesity has reached pandemic levels. Obesity often leads to insulin resistance, where the body’s response to insulin is diminished. GHRH/GH signaling is crucial in modulating insulin sensitivity as reduced GH levels due to impaired GHRH activity can exacerbate insulin resistance, contributing to the development of type 2 diabetes [91]. On the other hand, in adipocytes from morbidly obese subjects the expression of GHRH is higher than in non-obese subjects [42]. Furthermore, in adipocytes from patients with severe obesity, the expression of GHRHR and GHR is increased compared to patients without obesity. Additionally, GHRH has been shown to reduce adipocyte differentiation and increase lipolysis. These effects are mediated by GH and GHR [42].

In adipocytes, GHRH has been shown to inhibit lipid accumulation and promote lipolysis. Additionally, in vitro low doses of GHRH prevent the differentiation of human mesenchymal stem cells into adipocytes by reducing the expression of peroxisome proliferator-activated receptor γ (PPAR-γ), the key regulator of adipogenesis [42]. This contributes to maintaining energy balance.

GHRH influences lipid metabolism, and its dysregulation can lead to an abnormal lipid profile, such as increased low-density lipoprotein (LDL) cholesterol and decreased high-density lipoprotein (HDL) cholesterol [101]. This imbalance may contribute to the development of atherosclerosis, a key factor in cardiovascular diseases. In metabolic associated fatty liver disease (MAFLD), a study demonstrated a link between IGF-1 and IGFBPs and disease severity, as well as glycemia, with different IGFBPs playing distinct roles. IGFBP2 and IGFBP4 levels decreased, while IGFBP6 and IGFBP7 increased with greater steatosis. Hepatic IGFBP1 was inversely related to glycemia and insulin resistance, while IGFBP3 and IGFBP7 showed the opposite association. GHRH increased circulating IGFBP1 and IGFBP3, but lowered IGFBP2 and IGFBP6 [102]. Another study demonstrated a strong relationship between the GH axis, visceral adiposity index, and metabolic risk. A subset of seemingly healthy individuals exhibited some degree of visceral adipose dysfunction linked to GH and IGF-1 levels that fall short of meeting the criteria for overt GHD [103].

GHRH deficient mice are characterized by lower body weight, disproportionally high body fat accumulation, preferential metabolism of lipids compared to carbohydrates, improved insulin sensitivity, and extended lifespan [104–106]. Similar results were observed when the GHRH-deficient mice were fed a high-fat diet (HFD). The GHRH-KO mice receiving the HFD displayed respiratory exchange ratios and glucose oxidation rates comparable to GHRH-KO mice fed a control diet, while wild type mice fed an HFD showed significant reductions in these parameters [107]. This suggests that GH deficiency provides protection against the negative effects of diet-induced obesity in later life. Furthermore, in GHRH knockout mice, a significant reduction in serum lipids and liver ceramides, which are linked to aging-related tissue defects, was observed. Gene expression analysis revealed decreased ceramide synthesis in these mice, suggesting transcriptional changes contribute to this effect. These findings highlight a novel connection between GH deficiency, ceramide metabolism, and lifespan extension [108].

Patients with GHD are often moderately obese, characterized by increased body fat, particularly visceral fat. This suggests a connection between GH secretion and body fat levels [109]. Obesity can lead to feedback inhibition of pituitary GH secretion due to increased free fatty acid levels [110]. Additionally, insulin resistance prompts a compensatory rise in liver production of IGF-1, which remains stable in these patients [110]. This rise in IGF-1 may further suppress GH secretion through negative feedback mechanisms. Furthermore, the GH/IGF-1 axis is affected by glucose levels, with obese patients showing impaired GH secretion in response to hypoglycemia, indicating potential issues with hypothalamic function [111].

### Treatment of metabolic disorders with GHRH analogs

GHRH agonists and antagonists are analogs of native human GHRH with chemically modified amino acid sequences. Nearly 100 analogs with N- and C-terminal modifications of the GHRH(1–29)NH_2_ backbone have been developed [112]. Several of these analogs have shown promising results in vitro and in animal models in managing metabolic disorders, particularly by affecting pancreatic β-cells (Fig. 3; Tables 1 and 2).

**Table 1:** Metabolic impacts of GHRH agonists

	Experiment	Outcome	Ref
**GHRH agonists approved for clinical use**			
Tesamorelin	Patients with type 2 diabetes	Decrease of cholesterol and LDL	[134]
Tesamorelin	Patients with obesity and reduced GH secretion	Increased IGF-1, reduced visceral adipose tissue	[145, 146]
**GHRH agonists tested** ***in vitro*** **and in animal experiments**			
MR-502	Rat INS-1 cells	Increased insulin secretion	[41]
MR-409	Vascular calcification in db/db mice	Attenuation of vascular calcification	[136]
MR-409	STZ induced diabetes in mice	Improved glucose homeostasis, increased insulin levels, preservation of β-cell mass	[41, 113]
MR-409	Isolated rodent and human islets, mouse MIN6 cells	Decrease in β-cell death, improved insulin secretory function	[113]
MR-409	Rat INS-1 cells	Decreased ROS production, modulates Bcl-2, increases insulin, increased proliferation, Activation of ERK and AKT pathways, CREB phosphorylation	[41, 114]
MR-409	Pretreatment of rat islets before transplantation of mice with STZ-induced diabetes, treatment of mice post transplantation.	Increase in engraftment efficiency, increase in body weight, improved glucose homeostasis, increased insulin levels	[41]
MR-409	Rat and human hepatocytes, mice	Decrease in *Igf1/IGF1* mRNA, decrease in serum IGF-1	[115]
MR-403	Rat islets for transplantation	Increase cell survival and proliferation	[39]
MR-356	Rat INS-1 cells	Activation of ERK and AKT pathways	[41]
MR-356	Rat and human hepatocytes, mice	Decrease in *Igf1/IGF1* mRNA, decrease in serum IGF-1	[115]
JI-36	Rat INS-1 cells	Reduce apoptosis, increase proliferation	[120]
JI-36	Rat islets for transplantation	Increase cell survival and proliferation	[40, 132]

**Table 2:** Metabolic impacts of GHRH antagonists

	Experiment	Outcome	Ref
**GHRH antagonists tested in vitro and in animal experiments**			
MIA-690	Food intake in mice	Increased food intake and body weight, decreased leptin mRNA lvels	[137]
MIA-602	Rat INS-1 cells	Blocks agonist-induced cell survival	[120]
MIA-602	STZ induced diabetes in rats	Restoration of GLP-1 levels, reduced hyperglucagonemia	[121]

MR-409 is the agonist that has demonstrated the most significant effect on β-cells both in vitro and in vivo in animal experiments. When apoptosis was induced in isolated rodent or human islets by proinflammatory cytokines, treatment with MR-409, significantly enhanced their survival. Additionally, insulin secretion was enhanced [113]. In mice with low-dose streptozotocin- (STZ)-induced type 1 diabetes, the group treated with subcutaneous injections of MR-409, exhibited a greater β-cell mass. Furthermore, these mice exhibited better glucose homeostasis and higher insulin levels [113].

MR-409 functions by binding to GHRHR, triggering a signaling cascade that activates cAMP through adenylyl cyclase. This, in turn, leads to the activation of protein kinase A, which phosphorylates the transcription factor CREB at Ser133. This phosphorylation induces insulin receptor substrate 2 (IRS2) transcription and translation, which then activates AKT and ERK [41, 113]. Furthermore, in rat INS-1 cells treated with MR-409, it was demonstrated that CREB inhibits apoptosis by upregulating anti-apoptotic molecules like Bcl-2 and reducing reactive oxygen species (ROS) production, ultimately enhancing cell survival [114].

Overall, MR-409’s ability to protect and enhance the function of β-cells addresses two key issues in diabetes: the loss of β cells and the impairment of insulin secretion, thereby helping to maintain glucose homeostasis. These results suggest that MR-409 can potentially be used for treatment by preserving β-cell mass under proinflammatory conditions.

Treatment of rat INS-1 cells with another GHRH agonist MR-502 led to increased insulin secretion, while treatment with MR-356 was shown to activate ERK and AKT pathways [41].

The agonists MR-356 and MR-409 were shown to reduce the expression of IGF-1 in hepatocytes both in vitro and in vivo [115]. Additionally, GHRH agonists exhibit antioxidant properties [114, 116] and possess anti-inflammatory effects [116–118]. These characteristics suggest potential benefits of using GHRH agonists in early type 1 diabetes. This stage is marked by inflammatory cell infiltration, proinflammatory cytokine release, and β-cell destruction [90, 119].

In INS-1 cells, the GHRH antagonist MIA-602 was shown to block agonist-induced cell survival [120]. In a rat model of type 1 diabetes, it was demonstrated that GHRH receptors are upregulated in the small intestine [121]. When MIA-602 was administered subcutaneously to the rats, impaired GLP-1 levels were restored, and dyslipidemia and hyperglucagonemia were blunted [121] suggesting that antagonizing GHRH signalling in type 1 diabetes may improve GLP-1 function in the intestine.

As mentioned in the introduction, both allogenic [122] and xenogeneic [123–125] transplantation of the entire pancreas or isolated pancreatic islets hold promise for treating type 1 diabetes. Additionally, cellular regenerative therapies utilizing pluripotent stem cell-derived pancreatic β-cells [126–128] offer potential avenues for a curative approach. A major determinant for a successful outcome is the initial number of β-cells or the islet mass transplanted. Efficient islet isolation procedures and measures to minimize islet loss are therefore crucial [9, 129]. For instance, human pancreatic islets are susceptible to ferroptosis, a specific form of cell death [130, 131]. Thus, enhancing the survival of isolated islets or stem cell-derived β-cells could provide a significant advantage. Previous research has shown a substantial improvement in islet transplantation effectiveness when islets were preconditioned with GHRH agonists such as JI-36 [40, 132], MR-403 [39], and MR-409 [41]. This improved engraftment can be attributed, at least in part, to the increased secretion of IGF-1 and vascular endothelial growth factor [41].

Dyslipidemia, characterized by abnormal levels of lipids in the blood, is a major risk factor for atherosclerotic cardiovascular disease [133]. Tesamorelin, the only GHRH agonist used in clinical practice to date, has been shown to reduce serum total cholesterol and non-HDL cholesterol levels in individuals with type 2 diabetes [134]. However, administration of the GHRH analog tesamorelin to type 2 diabetes patients showed no changes in insulin response or diabetes control, despite decreasing cholesterol levels [134], suggesting that the effect is independent of the GHRH/GH/IGF-1 axis. This is consistent with data from animal studies using other GHRH analogs (Table 1), which also showed that the effect of subcutaneous administration of GHRH analogs is organ-specific and independent of the GHRH/GH/IGF-1 axis [23, 40, 121, 135].

Vascular calcification is a prevalent complication in type 2 diabetes, and unfortunately, there is currently no effective treatment available. When diabetic db/db mice were injected subcutaneously every day for 8 weeks with the GHRH agonist MR-409, vascular and heart calcification were attenuated without significantly affecting the pituitary GHRH/GH axis [136]. Thus, GHRH agonists might represent a new pharmacological strategy for treatment of diabetes-associated cardiovascular complications.

The effects of chronic subcutaneous administration of MR-409 and MIA-690 on feeding behavior and energy metabolism, were investigated in mice. Compared to vehicle, the GHRH agonist MR-409 had no effect, while the antagonist MIA-690 increased food intake and body weight [137]. Consequently, simultaneous stimulation of multiple receptor targets represents a logical strategy to amplify the insulinotropic effects of anti-diabetic drugs [138].

Adipose tissue dysfunction in obesity has been associated with impaired metabolic health and altered hormone release, including leptin, which plays a role in both obesity and metabolic disorders [139]. Leptin has been shown to influence GH release from the pituitary and may directly affect the development of hypothalamic GHRH neurons in the early postnatal period, suggesting that leptin signaling could impact linear growth during this time [140]. Additionally, the GHRH antagonist MIA-690 has been found to increase food intake in mice by lowering leptin mRNA levels in visceral fat [137]. Notably, the gut hormone ghrelin works synergistically with GHRH to enhance GH response [141], and GHRH has been identified as an allosteric modulator of the ghrelin receptor, while GHRH antagonists inhibit this effect [142]. These observations imply that GHRH may play a role in the various physiological effects of ghrelin, including the stimulation of GH secretion, food intake, and the regulation of energy balance.

In patients with GHD, GH therapy can improve body composition, reduce fat mass, increase lean body mass, and improve lipid profiles, highlighting the therapeutic potential of targeting GHRH/GH pathways in metabolic disorders [143]. Patients infected with human immunodeficiency virus (HIV) have a high prevalence of MAFLD often associated with increased visceral adipose tissue. The GHRH analog, tesamorelin, modified from GHRH(1–44)NH_2_ with a hexenoyl moiety attached to the amino-terminal tyrosine [144], has been shown to reduce visceral adipose tissue and to increase GH and IGF-1 levels in obese subjects with relative reductions in GH secretory capacity [145, 146]. Furthermore, tesamorelin was shown to reduce liver fat and prevent fibrosis progression in HIV-associated MAFLD [147–149]. Consequently, tesamorelin was approved in 2010 by the FDA for treatment of visceral adiposity in individuals with HIV-associated abdominal fat accumulation expecting lipodystrophy [150].

## Conclusion

GHRH plays a crucial role in metabolism, not only through its systemic function within the GHRH/GH/IGF-1 axis but also due to its extrapituitary effects on various endocrine organs. Preserving the remaining β-cell mass and supporting β-cell regeneration may offer alternative strategies for treating type 1 diabetes. GHRH agonists have been shown to promote β-cell proliferation and survival in both in vitro studies and animal models of diabetes and transplantation, indicating their potential as an alternative approach. However, to date, only the GHRH analog tesamorelin has been tested in clinical settings and approved by the FDA. Further research in humans is necessary to explore the therapeutic potential of additional GHRH agonists and antagonists, as well as their potential side effects.

## Data Availability

No datasets were generated or analysed during the current study.

## Abbreviations

cAMP: Cyclic adenosine monophosphate
CNS: Central nervous system
GH: Growth hormone
GHD: Growth hormone deficiency
GHR: Growth hormone receptor
GHRH: Growth hormone-releasing hormone
GHRHR: Growth hormone-releasing hormone receptor
GLP-1: Glucagon-like peptide 1
HDL: High-density lipoprotein
HFD: High fat diet
HIV: Human immunodeficiency virus
IGF-1: Insulin-like growth factor-1
IGF-1R: Insulin-like growth factor-1 receptor
IGFBP: IGF-binding proteins
IRS2: Insulin receptor substrate 2
JAK: Janus kinase
LDL: Low-density lipoprotein
MAFLD: Metabolic associated fatty liver disease
MAPK: Mitogen activated protein kinase
PACAP: Pituitary adenylate-cyclase-activating polypeptide
PYY: Peptide YY
PI3K: Phosphatidylinositol 3’-kinase
PKA: Protein kinase A
ROS: Reactive oxygen species
Stat: Signal transducers and activators of transcription
STZ: Streptozotocin
VIP: Vasoactive intestinal polypeptide
